# Sex differences in systemic metabolites at four life stages: cohort study with repeated metabolomics

**DOI:** 10.1186/s12916-021-01929-2

**Published:** 2021-02-24

**Authors:** Joshua A. Bell, Diana L. Santos Ferreira, Abigail Fraser, Ana Luiza G. Soares, Laura D. Howe, Deborah A. Lawlor, David Carslake, George Davey Smith, Linda M. O’Keeffe

**Affiliations:** 1 MRC Integrative Epidemiology Unit at the University of Bristol, Oakfield House, Bristol, BS8 2BN UK; 2 Population Health Sciences, Bristol Medical School, University of Bristol, Bristol, UK; 3 Bristol NIHR Biomedical Research Centre, Bristol, UK; 4 School of Public Health, Western Gateway Building, University College Cork, Cork, Ireland

**Keywords:** Sex differences, Metabolites, Coronary heart disease, NMR metabolomics, Epidemiology, ALSPAC

## Abstract

**Background:**

Males experience higher rates of coronary heart disease (CHD) than females, but the circulating traits underpinning this difference are poorly understood. We examined sex differences in systemic metabolites measured at four life stages, spanning childhood to middle adulthood.

**Methods:**

Data were from the Avon Longitudinal Study of Parents and Children (7727 offspring, 49% male; and 6500 parents, 29% male). Proton nuclear magnetic resonance (^1^H-NMR) spectroscopy from a targeted metabolomics platform was performed on EDTA-plasma or serum samples to quantify 229 systemic metabolites (including lipoprotein-subclass-specific lipids, pre-glycaemic factors, and inflammatory glycoprotein acetyls). Metabolites were measured in the same offspring once in childhood (mean age 8 years), twice in adolescence (16 years and 18 years) and once in early adulthood (25 years), and in their parents once in middle adulthood (50 years). Linear regression models estimated differences in metabolites for males versus females on each occasion (serial cross-sectional associations).

**Results:**

At 8 years, total lipids in very-low-density lipoproteins (VLDL) were lower in males; levels were higher in males at 16 years and higher still by 18 years and 50 years (among parents) for medium-or-larger subclasses. Larger sex differences at older ages were most pronounced for VLDL triglycerides—males had 0.19 standard deviations (SD) (95% CI = 0.12, 0.26) higher at 18 years, 0.50 SD (95% CI = 0.42, 0.57) higher at 25 years, and 0.62 SD (95% CI = 0.55, 0.68) higher at 50 years. Low-density lipoprotein (LDL) cholesterol, apolipoprotein-B, and glycoprotein acetyls were generally lower in males across ages. The direction and magnitude of effects were largely unchanged when adjusting for body mass index measured at the time of metabolite assessment on each occasion.

**Conclusions:**

Our results suggest that males begin to have higher VLDL triglyceride levels in adolescence, with larger sex differences at older ages. Sex differences in other CHD-relevant metabolites, including LDL cholesterol, show the opposite pattern with age, with higher levels among females. Such life course trends may inform causal analyses with clinical endpoints in specifying traits which underpin higher age-adjusted CHD rates commonly seen among males.

**Supplementary Information:**

The online version contains supplementary material available at 10.1186/s12916-021-01929-2.

## Background

Coronary heart disease (CHD) remains the leading cause of death globally [1, 2]. Recent decades have seen age-adjusted incidence and mortality rates decline substantially in higher-income countries [3], but those declines are now slowing [4, 5] and total numbers of cases are rising in most countries owing to population ageing and growth [2, 6]. Age-adjusted CHD rates are higher among males than females [7], and reasons for this are gaining clarity. For example, males are known to store more fat in visceral and ectopic compartments which drives insulin resistance [8, 9] and results in higher type 2 diabetes rates among males [10]. Males also have higher systolic blood pressure than females from adolescence to middle adulthood, although this difference narrows or even reverses in older age [11–13].

Sex differences in circulating lipids are more contradictory. Adult females tend to have lower triglyceride levels compared with adult males, potentially due to hormonal mechanisms [14, 15], yet adult females also tend to have higher cholesterol in low-density lipoprotein (LDL) particles [12, 16]. Such comparisons have been based mostly on circulating traits measured by conventional clinical assays. More detailed measures from targeted metabolomic platforms now exist [17] which have helped characterise the cardiometabolic profile of pregnancy and menopause [18, 19]. Knowledge of sex differences in these more detailed traits at multiple life stages may help reveal more specific circulating pathways that underpin sex differences in age-adjusted rates of CHD, but no such investigation has yet been conducted.

We aimed in this study to better characterise sex differences in CHD-relevant metabolites at multiple life stages, to help identify circulating traits that may underpin known sex differences in CHD burden. Using data from a multi-generational pregnancy cohort study, we estimated the total effect of biological sex on over 200 systemic metabolites quantified using targeted metabolomics on EDTA-plasma or serum samples, including lipoprotein subclass-specific cholesterol and triglycerides, amino acids, and inflammatory glycoprotein acetyls, at four life stages. These metabolites were measured once in childhood (mean age 8 years), twice in adolescence (16 years and 18 years), and once in early adulthood (25 years) on the same male and female offspring (Generation-1 (G1)), as well as on their parents (Generation-0 (G0)) in middle adulthood (50 years). We also examined the extent to which adiposity may mediate any total effect of sex on metabolites by adjusting for body mass index (BMI) at each life stage.

## Methods

### Study population

Data were from the Avon Longitudinal Study of Parents and Children (ALSPAC), a population-based birth cohort study in which 14,541 pregnant women expected to deliver between 1 April 1991 and 31 December 1992 were recruited from the former county of Avon in southwest England. Offspring (G1 cohort) alive at 1 year (*n* = 13,988) have since been followed up with multiple assessments with an additional 913 children enrolled over the course of the study [20, 21]. Mothers and fathers (termed hereafter as ‘partners’ as not all are biological fathers) of offspring participants have also been followed with multiple assessments (G0 cohort) [22]. Parental data used here were primarily from mothers who attended a clinic assessment between December 2008 and July 2011 and from partners who attended a clinic assessment between September 2011 and February 2013.

The study website contains details of all the data that is available through a fully searchable data dictionary and variable search tool (http://www.bristol.ac.uk/alspac/researchers/our-data/).

### Assessment of systemic metabolites

Among offspring (G1), blood samples were drawn in clinics at mean (standard deviation, SD) ages 7.5 years (0.3 years), 15.4 years (0.3 years), 17.7 years (0.4 years), and 24.5 years (0.8 years). Proton nuclear magnetic resonance (^1^H-NMR) spectroscopy from a targeted metabolomics platform [23] was performed on EDTA-plasma samples from each of these four occasions to quantify 229 metabolites (149 concentrations plus 80 ratios derived from these) including cholesterol, triglyceride, and other lipid content in lipoprotein subclass particles (very-low-density lipoprotein (VLDL), intermediate-density lipoprotein (IDL), low-density lipoprotein (LDL), high-density lipoprotein (HDL)), apolipoprotein-B and apolipoprotein-A-1, fatty acids and amino acids, and inflammatory glycoprotein acetyls. Bloods at age 8 years were taken while not fasting and bloods at age 16 years, 18 years, and 25 years were taken after a minimum of a 6-h fast (stability in these metabolite concentrations has been shown over different fasting durations [24]).

Among parents (G0), blood samples were drawn in clinics at mean (SD) age 47.9 years (4.5 years) among mothers and 53.3 years (5.4 years) among partners. The same ^1^H-NMR metabolomics platform used among G1 offspring was performed on serum samples taken on G0 parents after a minimum of a 6-h fast during these clinics to quantify the same 229 metabolites described above. Among mothers only, additional blood samples were available from visits conducted between July 2011 and June 2013 when mothers were of mean (SD) age 50.8 years (4.4 years), from which additional measures of the same 229 metabolites were quantified with NMR as prior. If mothers were missing data on all metabolites on the first measurement occasion but had data on at least one metabolite on the second measurement occasion, then metabolite values from that second occasion were used to replace missing values on the first occasion. The number of mothers with these replacements ranged from 215 to 223 across metabolites.

### Participants eligible for analyses

**Fig. 1:**
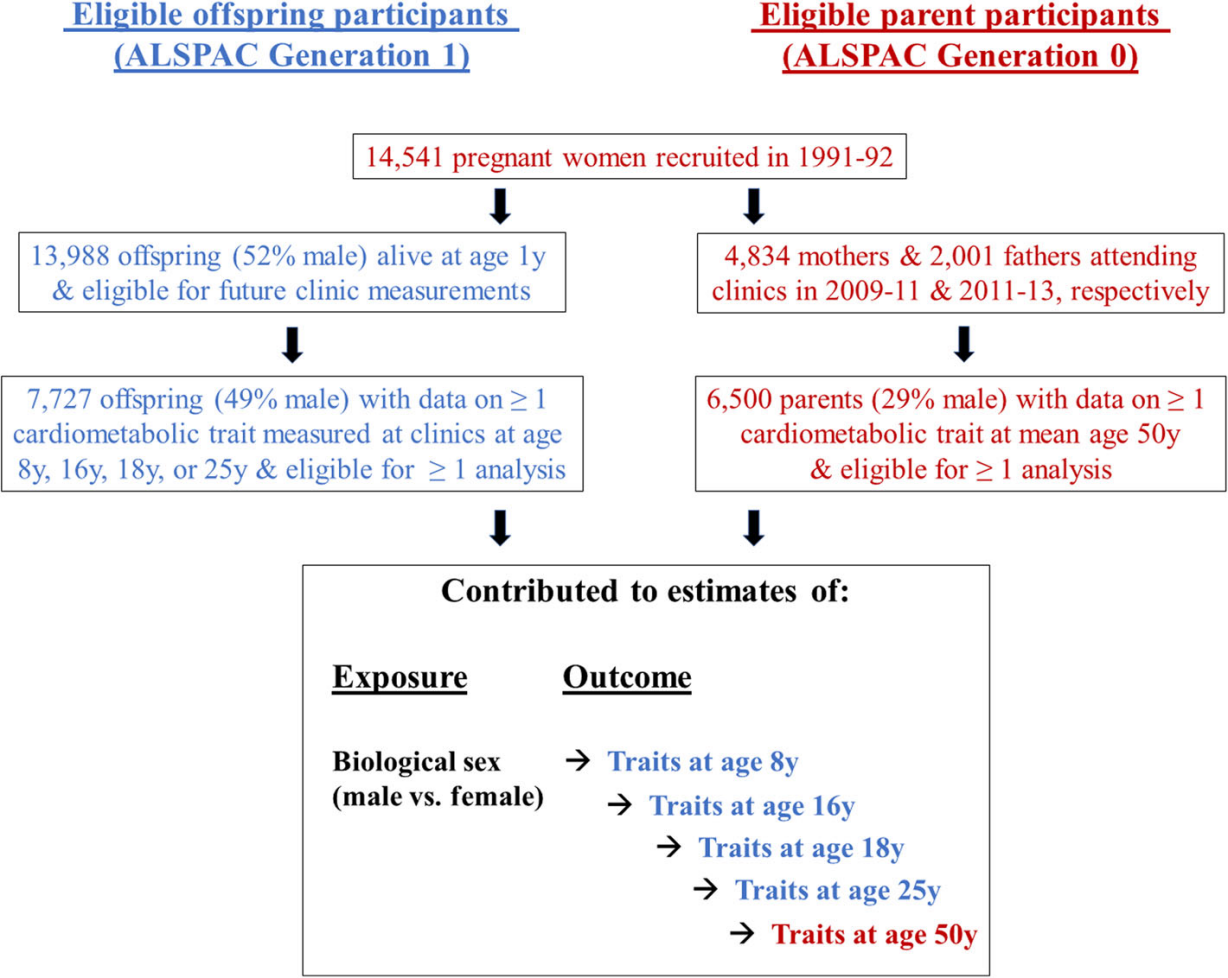
Selection of participants eligible for inclusion in at least one analysis

To allow full use of measured data, analyses were conducted using maximum numbers of participants (with N varying across ages and between metabolites). Participants were eligible for inclusion in analyses at any age if they had data on sex, age, and at least one of the metabolites. This resulted in 7727 eligible G1 offspring (3763 males, 3964 females; Fig. 1) contributing to some analyses, with sample sizes for age-specific analyses ranging from 5403 to 5515 at age 8 years, from 3162 to 3358 at age 16 years, from 3090 to 3174 at age 18 years, and from 3204 to 3260 at age 25 years. The single time-point analysis in G0 parents included 6500 eligible parents (1855 males, 4645 females; Fig. 1), with sample sizes for each metabolite ranging between 5800 and 6500. No specific exclusions were made based on cardiometabolic diagnoses or medication use in G1 or G0.

### Statistical approach

We examined several characteristics of participants eligible for inclusion in analyses by sex; these are described in Additional file 1: Supplementary Methods [18, 25–28]. These characteristics were also examined among participants who were not eligible for inclusion in our analyses, defined as not having data on sex, age, and ≥ 1 metabolite at any measurement occasion (6085 G1s and 7811 G0s) to examine potential for selection bias. We additionally examined characteristics in G0 females, G1 males and G1 females by the participation status of G0 males (mothers’ partners), to further assess selection bias.

Metabolites at each measurement occasion were standardised into SD units using z-scores (subtracting the sex-combined mean and dividing by the sex-combined SD). Linear regression models with robust standard errors (to accommodate skewed outcome distributions) were used to examine the mean difference and 95% confidence interval (CI) for the association of sex with each standardised metabolite on each occasion, adjusting for age at the time of metabolite assessment. Given differences in the age of G0 parents, we centred age at 50 years and included an interaction term between sex and centred age to allow associations of sex with metabolites to differ by age. Estimates are therefore interpreted as the difference in mean (in SD units) of each metabolite for males compared with females.

**Fig. 2:**
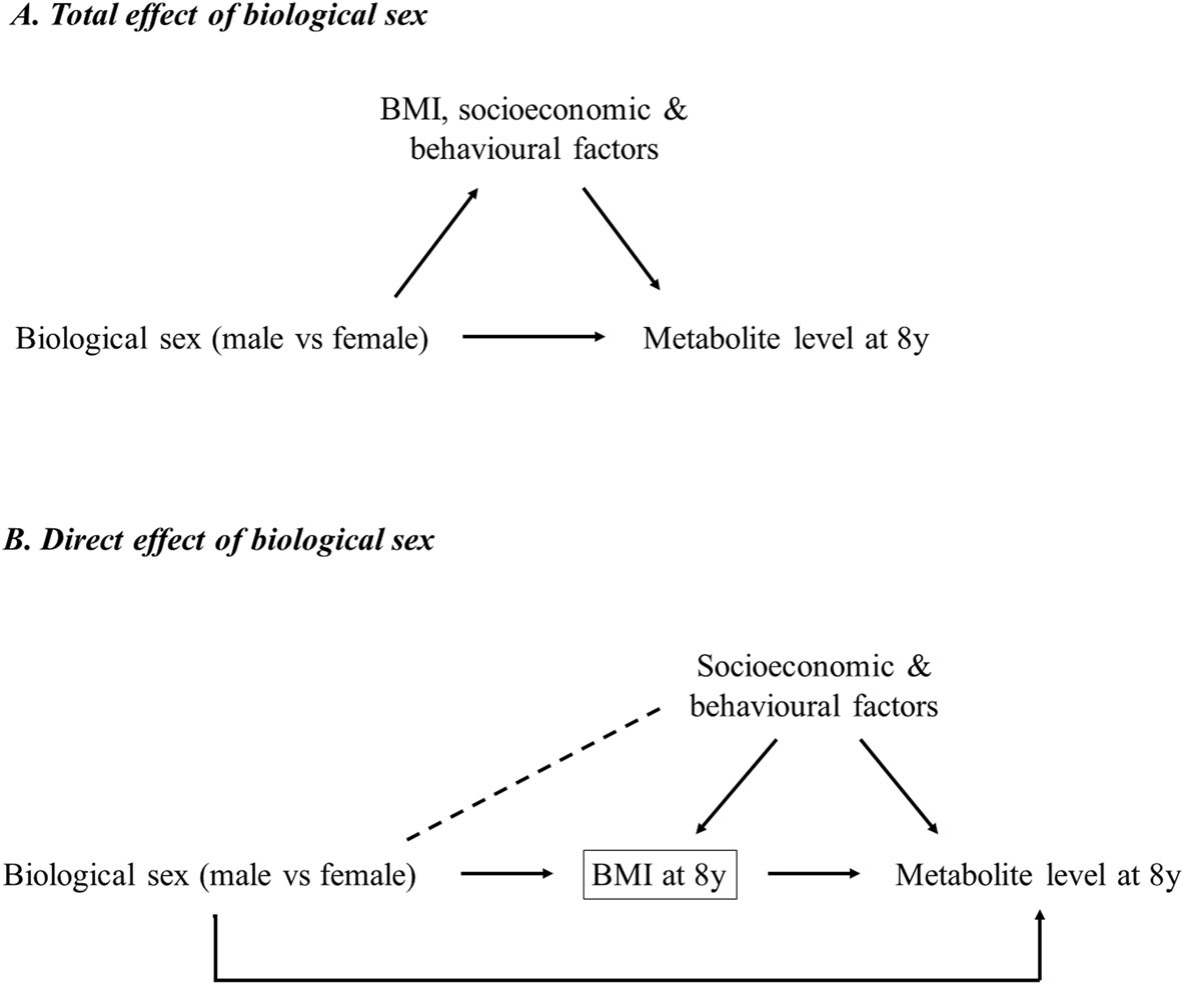
Illustration of the potential for collider bias in analyses of biological sex and metabolite levels when adjusting for BMI. **a** Estimates of the total effect of biological sex (male vs female: exposure of interest) on metabolite levels at each time point (shown here is 8 years for example) are not prone to confounding by BMI, socioeconomic, or behavioural factors because whilst such factors could influence metabolite levels, they could not plausibly influence biological sex assignment at conception and would be considered mediators of effect. **b** Conditioning on BMI (potential mediator) measured at the time of metabolite assessment could produce estimates of a direct effect of sex on metabolite levels at each time point, but this could also induce non-causal associations between sex and metabolite levels due to an induced association between sex and socioeconomic or behavioural factors (confounders of the mediator-outcome associations). Such bias may be correctable via adjustment for confounders of the mediator-outcome association, but these could be time-varying and unmeasured. We considered the potential for collider bias to be low here given the strong agreement between effect estimates of BMI with these same metabolites from several observational and Mendelian randomisation analyses (i.e. low potential for mediator-outcome confounding) [29–32]

In the first set of models, no adjustments were made for BMI or lifestyle-related factors because, although such factors likely influence metabolites, they cannot influence biological sex (male vs female: the exposure of interest) and thus cannot be confounding factors of the total effects of sex on metabolites under study (such factors would be mediators). To examine the extent to which adiposity may mediate any total effect of sex on metabolites, we repeated the analyses mentioned above with additional adjustment for BMI at the time of metabolite measurement. Each BMI measure was centred on its sex-specific mean value, and an interaction term between sex and centred BMI was included to allow effects of sex on metabolites to differ by BMI. Conditioning on mediators could induce collider bias through (un) measured confounders of the mediator-outcome association (illustrated in Fig. 2); we considered the potential for this bias to be low here given the strong agreement between effect estimates of BMI with these same metabolites across several observational and Mendelian randomisation analyses (i.e. low potential for mediator-outcome confounding) [29–32].

All models were additionally run using original (non-SD; mostly mmol/l) units to aid clinical interpretation. Meta-regression was used to examine whether effect estimates change linearly with mean age on each occasion, via *P* values for trend across occasions. This was applied to directly test heterogeneity in estimates across offspring and parents (analysed in separate datasets) under an independence assumption.

We performed several supplementary analyses to examine how G0 mothers’ menopausal status and overall sampling strategies influence results; these are described in Additional file 1: Supplementary Methods.

Because our statistical aims involve estimation, we present exact *P* values and focus on effect size and precision, as recommended [33, 34]. Analyses were conducted using Stata 15.1 (StataCorp, College Station, TX, USA).

## Results

### Sample characteristics

Male and female G1s had a similar mean age on each occasion—mean (SD) age overall was 7.5 years (0.3 years), 15.5 years (0.3 years), 17.8 years (0.4 years), and 24.5 years (0.8 years) successively (Table 1). A minority of males and females (each < 5.0%) were of a non-white ethnicity. Maternal and partner education levels were similar among male and female G1s, as was the prevalence of maternal and partner smoking during/around pregnancy. Mean (SD) age at peak height velocity among G1s was 12.6 years (1.3 years) overall; this was later among males than females at 13.6 years (0.9 years) and 11.7 years (0.8 years), respectively. Based on this, 4.5% of male and 5.7% of female G1s were post-pubertal on the first measurement occasion (i.e. had reached peak height prior to the 8 years clinic), while 100% of females and 97% of males were post-pubertal on the second measurement occasion (i.e. had reached peak height prior to the 16 years clinic).

**Table 1:** Characteristics of ALSPAC G1 offspring and G0 parents eligible for analyses

		G1 offspring				G0 parents		
		***Males***		***Females***		***Males***		***Females***
***Characteristics***	***N***		***N***		***N***		***N***	
Age (years) on first clinic occasion—mean (SD)	3410	7.5 (0.3)	3376	7.5 (0.3)	1855	53.2 (5.3)	4645	47.9 (4.5)
Non-white ethnicity—% (*N*)	3328	< 5.0 (NA*)	3422	< 5.0 (NA*)	4175	< 5.0 (NA*)	4219	< 5.0 (NA*)
Highest maternal education—% (*N*)	3389		3470		–		4233	
Certificate of secondary education		13.3 (450)		14.0 (484)		–		10.2 (430)
Vocational		8.4 (286)		8.4 (293)		–		7.4 (313)
Ordinary level		35.7 (1209)		34.2 (1185)		–		34.3 (1453)
Advanced level		26.3 (891)		26.9 (932)		–		29.3 (1238)
Degree		16.3 (553)		16.6 (576)		–		18.9 (799)
Highest partner education—% (*N*)	3293		3378		4142		–	
Certificate of secondary education		19.0 (625)		21.5 (725)		16.9 (699)		–
Vocational		7.6 (251)		7.7 (259)		7.4 (308)		–
Ordinary level		22.3 (734)		21.4 (723)		21.0 (869)		–
Advanced level		28.1 (925)		27.4 (926)		29.3 (1213)		–
Degree		23.0 (758)		22.1 (745)		25.4 (1053)		–
BMI on first clinic occasion—mean (SD)	3385	16.1 (1.9)	3350	16.4 (2.2)	1831	27.5 (4.0)	4629	26.6 (5.3)
Maternal BMI pre-pregnancy—mean (SD)	3163	22.9 (3.8)	3246	22.8 (3.6)	–	–	3991	22.5 (3.4)
Partner BMI at pregnancy—mean (SD)	2438	25.1 (3.1)	2544	25.2 (3.3)	1493	24.7 (3.0)	–	–
Maternal smoking during pregnancy—% (*N*)	3133	22.5 (706)	3181	22.4 (713)	–	–	3919	17.0 (665)
Partner smoking during pregnancy—% (*N*)	3271	29.7 (972)	3333	31.6 (1054)	4070	27.3 (1112)		–
Age (y) at peak height velocity—mean (SD)	2404	13.6 (0.9)	2688	11.7 (0.8)	–	–	–	–
Menopause status (STRAW)—% (*N*)	–	–	–	–	–	–	3513	
Pre-menopause		–		–		–		62.1 (2181)
Peri-menopause		–		–		–		19.4 (680)
Post-menopause		–		–		–		18.6 (652)

Participants described are those with data on sex, age, and at least 1 metabolite on any measurement occasion. ‘Maternal/partner’ characteristics refer to own status among parents. *BMI* body mass index. *STRAW* Stages of Reproductive Age Workshop. *Cells have been censored due to small cell counts in accordance with study ethics

Male G0s (partners) were on average older than female G0s (mothers) at the time of assessment—mean (SD) age was 53.2 years (5.3 years) among males vs 47.9 years (4.5 years) among females (Table 1). A similarly low proportion of males and females (each < 5.0%) was non-white, while more males than females reported a degree as their highest qualification (25.4% vs. 18.9% respectively). Smoking during the pregnancy was more common among males than females (27.3% vs. 17.0%, respectively). Among mothers, 62.1% were pre-menopause by the time of clinic assessment; 19.4% were peri-menopause and 18.6% were post-menopause.

G1s who were ineligible for any analysis had a lower parental educational attainment than those who were eligible and were more likely to have parents who reported smoking during pregnancy (Additional file 2: Supplementary Table 1). G0 mothers whose partner did participate were similar to mothers whose partner did not participate on most characteristics, but mothers whose partner participated had a higher proportion of degree holders and a lower proportion smoking during pregnancy compared with mothers whose partners did not participate (Additional file 3: Supplementary Table 2). G1s whose mothers’ partners participated were similar to G1s whose mothers’ partners did not participate on most characteristics, but G1s whose mothers’ partners participated were less likely to be non-white, more likely to have parents with academic degrees, and less likely to have parents who smoked during pregnancy (Additional file 4: Supplementary Table 3). The magnitude of the difference by sex in these characteristics among G1s did not differ greatly by mothers’ partner participation status.

### Sex differences in lipid metabolites

**Fig. 3:**
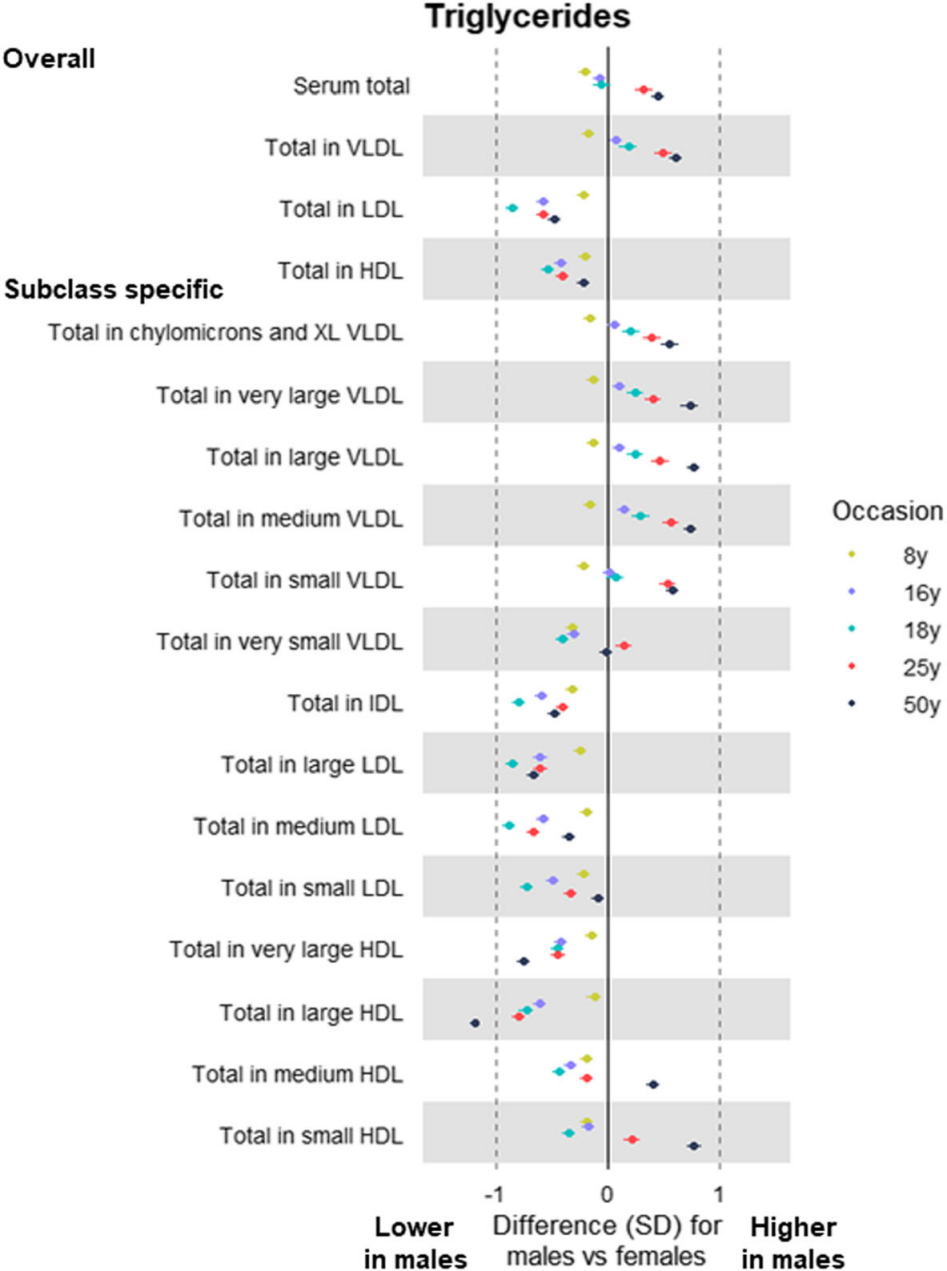
Sex differences in lipoprotein triglycerides at different life stages in ALSPAC. Metabolite measures at mean age 8 years, 16 years, 18 years, and 25 years are among ALSPAC G1 offspring; metabolite measures at mean age 50 years are among ALSPAC G0 parents (analysed separately). Offspring models are adjusted for age at metabolite assessment; parent models are adjusted for age and age-by-sex interaction. VLDL, very-low-density lipoprotein. IDL, intermediate-density lipoprotein. LDL, low-density lipoprotein. HDL, high-density lipoprotein

**Fig. 4:**
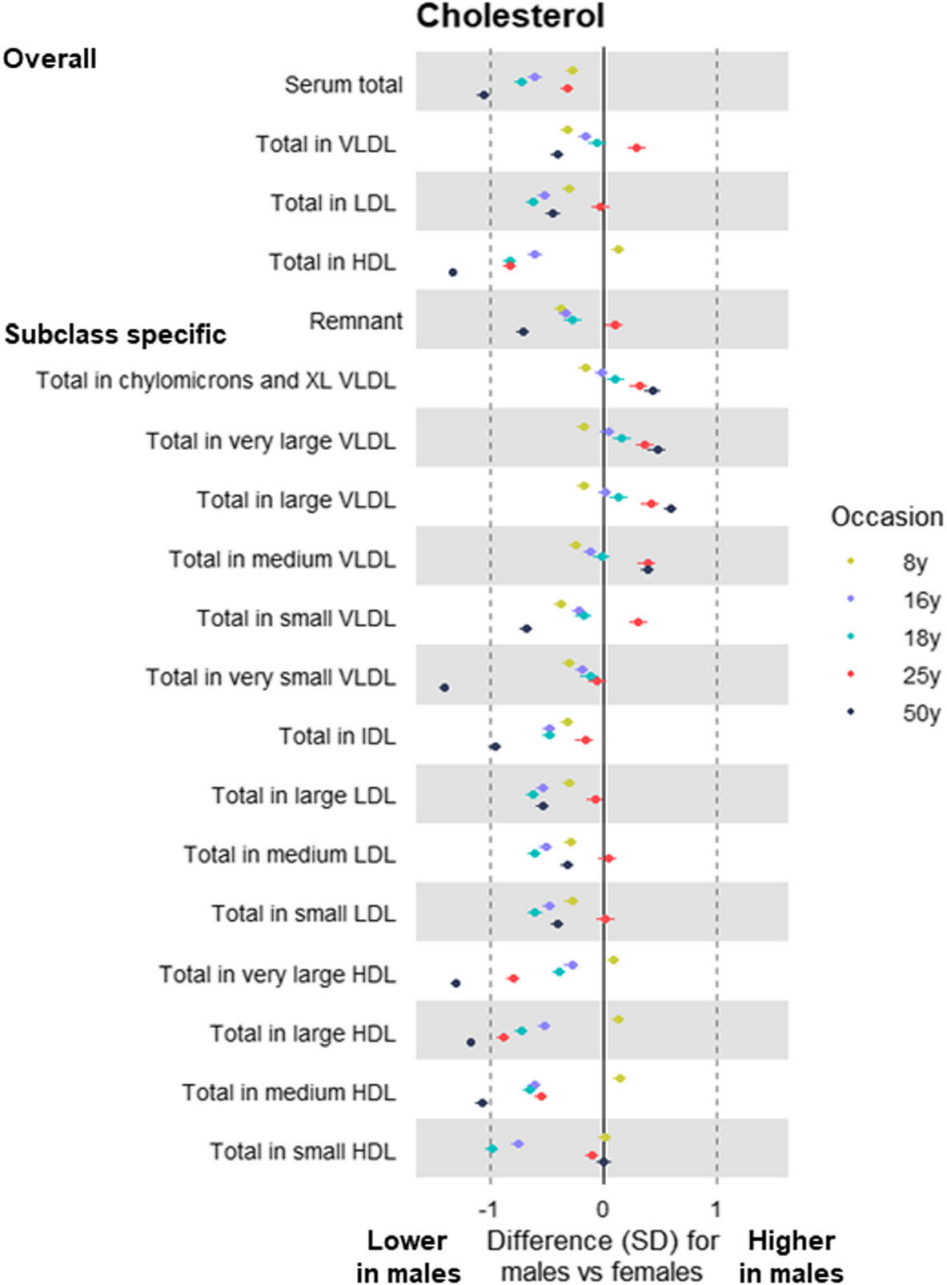
Sex differences in lipoprotein cholesterol at different life stages in ALSPAC. Metabolite measures at mean age 8 years, 16 years, 18 years, and 25 years are among ALSPAC G1 offspring; metabolite measures at mean age 50 years are among ALSPAC G0 parents (analysed separately). Offspring models are adjusted for age at metabolite assessment; parent models are adjusted for age and age-by-sex interaction. VLDL, very-low-density lipoprotein. IDL, intermediate-density lipoprotein. LDL, low-density lipoprotein. HDL, high-density lipoprotein

**Fig. 5:**
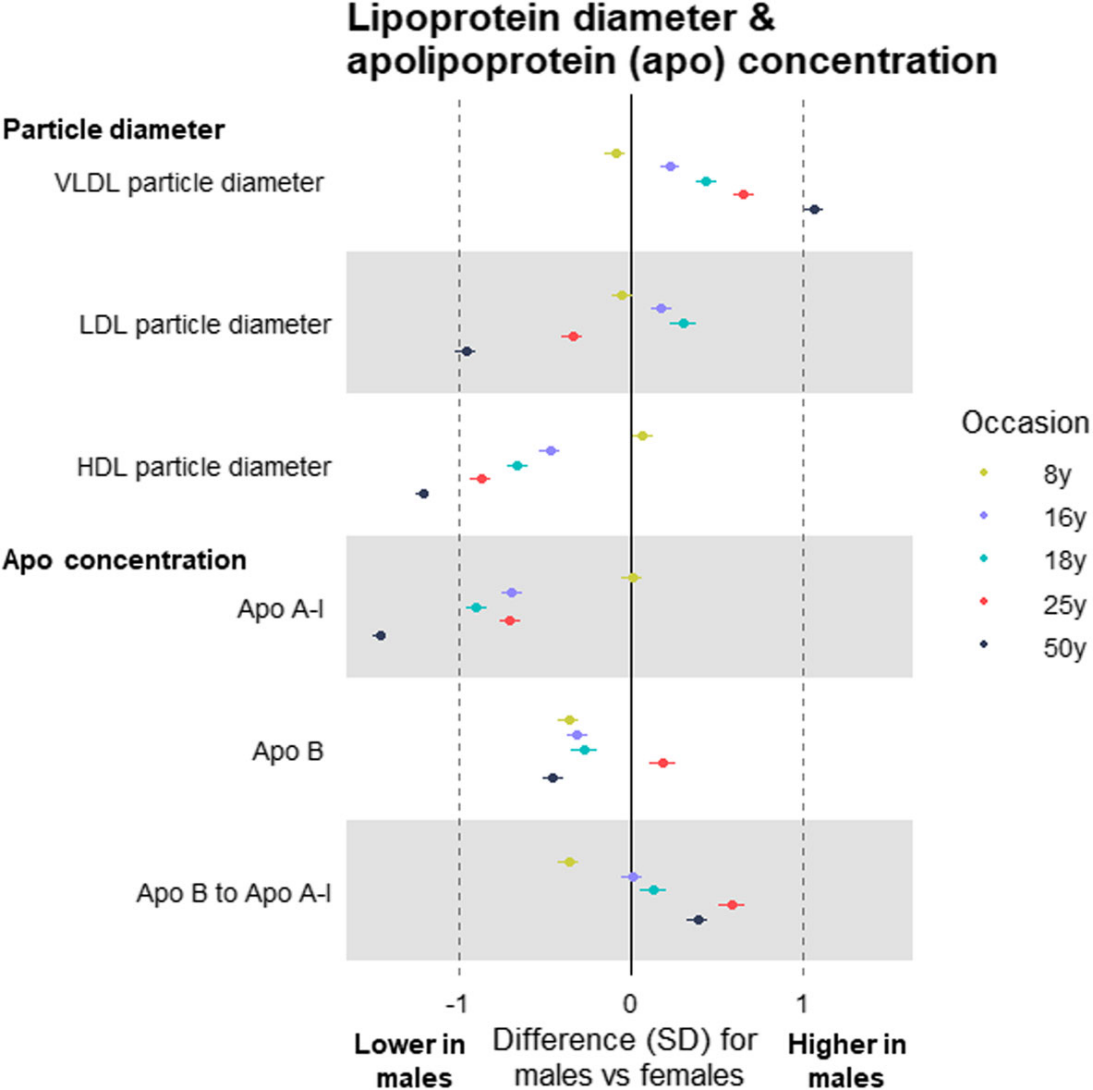
Sex differences in lipoprotein particle size and apolipoprotein concentration at different life stages in ALSPAC. Metabolite measures at mean age 8 years, 16 years, 18 years, and 25 years are among ALSPAC G1 offspring; metabolite measures at mean age 50 years are among ALSPAC G0 parents (analysed separately). Offspring models are adjusted for age at metabolite assessment; parent models are adjusted for age and age-by-sex interaction. VLDL, very-low-density lipoprotein. LDL, low-density lipoprotein. HDL, high-density lipoprotein

At 8 years, total lipids were lower among males than females in all lipoprotein subclasses including VLDL, IDL, and LDL, except for HDL subclasses in which total lipids were higher among males (Additional file 5: Supplementary Table 4; estimates in original/non-SD units in Additional file 6: Supplementary Table 5). At 16 years, levels of total lipids in (medium and larger) VLDL subclasses were similar between the sexes, but sex differences in these emerged at 18 years (among G1s) and were evident at 50 years (among G0s) for subclasses that were medium or larger—e.g. total lipids in large VLDL were higher among males than females by 0.21 SD (95% CI = 0.14, 0.28), by 0.45 SD (95% CI = 0.37, 0.52), and by 0.72 SD (95% CI = 0.65, 0.79) at 18 years, 25 years, and 50 years, respectively. *P* values for trend across occasions were generally lowest for lipids in VLDL, supporting patterns of higher levels at older ages—e.g. *P* = 0.02 for total lipids in large VLDL. The higher levels of VLDL lipids seen among males at older ages were most pronounced for triglycerides in VLDL (Fig. 3). Cholesterol was higher among males in large VLDL particles, but lower among males in other particles including LDL, with inconsistent sex differences at 25 years apart from cholesterol in HDL (Fig. 4). Sex differences in lipoprotein particle sizes themselves were larger at older ages—appearing higher among males for VLDL and lower among males for HDL (Fig. 5). Apolipoprotein-B was also notably lower among males at all ages apart from 25 years, while apolipoprotein-B as a function of apolipoprotein-A-1 was higher among males at older ages (Fig. 5). The direction and magnitude of effects were largely unchanged when adjusting for BMI measured at the time of metabolite assessment on each occasion. For example, when adjusting for BMI, total lipids in VLDL were lower in males by − 0.15 SD (95% CI = − 0.20, − 0.10) at 8 years but higher in males by 0.21 SD (95% CI = 0.14, 0.28), by 0.46 SD (95% CI = 0.39, 0.53), and by 0.67 SD (95% CI = 0.61, 0.73) at 18 years, 25 years, and 50 years, respectively (Additional file 7: Supplementary Table 6; estimates in original/non-SD units in Additional file 8: Supplementary Table 7).

### Sex differences in pre-glycaemic and inflammatory metabolites

**Fig. 6:**
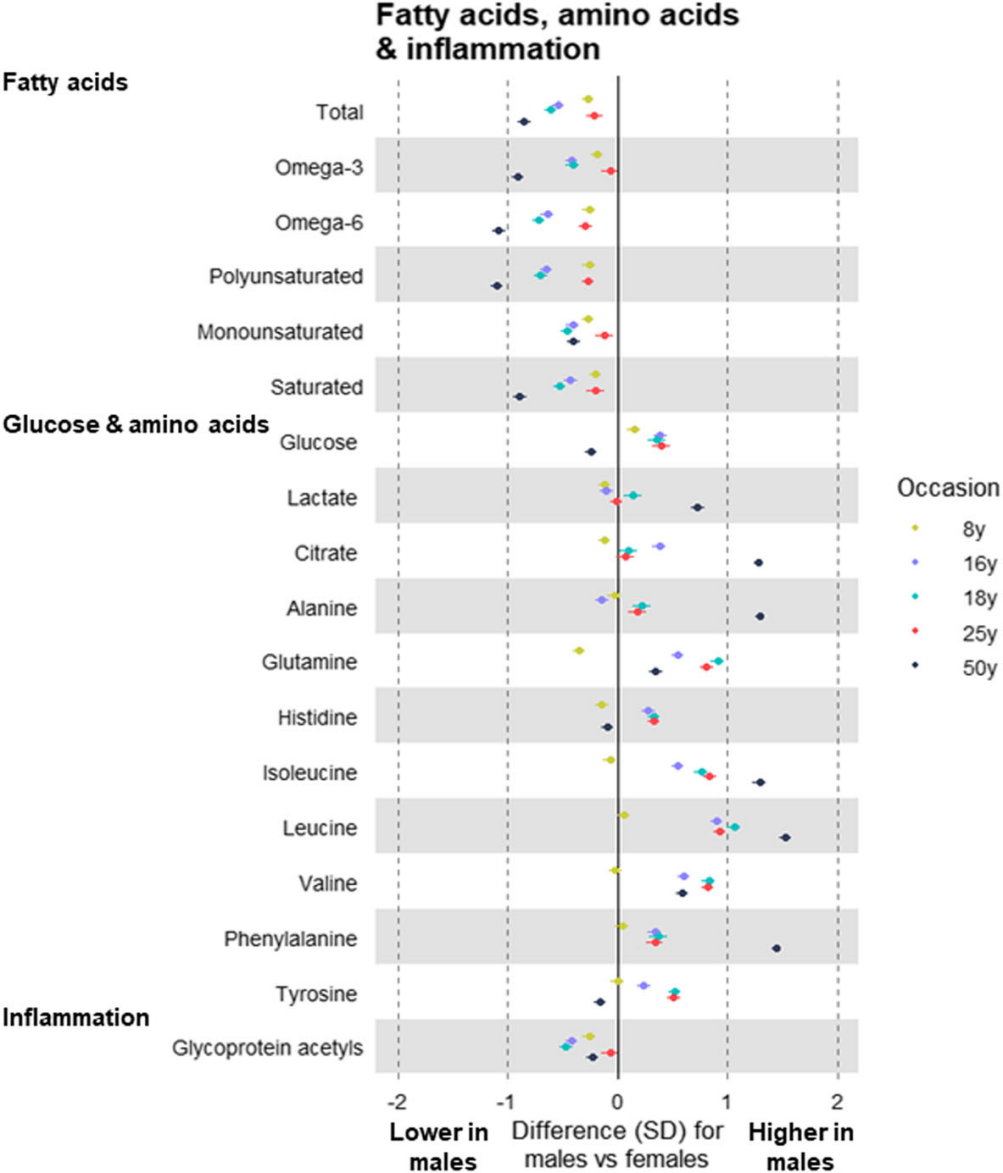
Sex differences in fatty acids, glycolysis-related metabolites, amino acids, and glycoprotein acetyls at different life stages in ALSPAC. Metabolite measures at mean age 8 years, 16 years, 18 years, and 25 years are among ALSPAC G1 offspring; metabolite measures at mean age 50 years are among ALSPAC G0 parents (analysed separately). Offspring models are adjusted for age at metabolite assessment; parent models are adjusted for age and age-by-sex interaction

Fatty acid levels tended to be lower among males than females, with some evidence that this difference was larger at older ages—e.g. males had lower polyunsaturated fatty acids by − 0.26 SD (95% CI = − 0.32, − 0.21) at 8 years among G1s and by − 1.10 SD (95% CI = − 1.16, − 1.05) at 50 years among G0s (Fig. 6; Additional file 5: Supplementary Table 4). Glucose was consistently higher among male G1s, whereas among G0s at 50 years glucose was − 0.24 SD (95% CI = − 0.30, − 0.17) lower among males. Lactate and citrate levels were markedly higher among males at 50 years, with sex differences of 0.73 SD (95% CI = 0.66, 0.79) and 1.28 SD (95% CI = 1.23, 1.32), respectively. Amino acids were consistently higher among males after 8 years, particularly branched chain amino acids—e.g. leucine was 0.06 SD (95% CI = 0.003, 0.11) higher among males at 8 years and 1.53 SD (95% CI = 1.47, 1.58) higher among males at 50 years. Comparably large sex differences were seen at 50 years for isoleucine (1.29 SD, 95% CI = 1.23, 1.34 higher among males) and for phenylalanine (1.44 SD, 95% CI = 1.40, 1.49 higher among males). The direction and magnitude of effects were again largely unchanged when adjusting for measured BMI at the time of metabolite assessment (Additional file 7: Supplementary Table 6).

Small sex differences were seen in ketone bodies at older ages. Creatinine was consistently higher among males after 8 years; this difference was largest at 25 years (1.24 SD, 95% CI = 1.18, 1.30) and was smaller at 50 years (0.51 SD, 95% CI = 0.45, 0.56; Additional file 5: Supplementary Table 4). Males had consistently lower glycoprotein acetyls with little evidence of trend across occasions (*P* = 0.62); this sex difference was smaller after 18 years (− 0.23 SD, 95% CI = − 0.29, − 0.16 at age 50 years without adjustment for BMI, and − 0.27 SD, 95% CI = − 0.33, − 0.21 with adjustment for BMI; Fig. 6).

### Supplementary analyses

When comparing middle-aged males with females who were pre-menopause, sex differences in metabolites were similar to main estimates (Additional file 9: Supplementary Table 8; non-SD estimates in Additional file 10: Supplementary Table 9). When comparing middle-aged males with females who were post-menopause, sex differences in metabolites were smaller, particularly for VLDL lipids—e.g. total lipids in large VLDL were 0.66 SD (95% CI = 0.58, 0.73) higher among males. In contrast, sex differences were larger for lipids in IDL, LDL, and HDL—e.g. total cholesterol in IDL was − 1.01 SD (95% CI = − 1.08, − 0.95) lower among males. Sex differences were smaller for branched chain amino acids, but were generally larger for fatty acids, glucose, and glycoprotein acetyls—e.g. − 0.36 SD (95% CI = − 0.44, − 0.29) lower among males for glycoprotein acetyls. The direction and magnitude of effects were again similar when adjusting for BMI (Additional files 11 and 12: Supplementary Tables 10 and 11).

Sex differences were similar when above sets of analyses were repeated using a complete case sample of 769 G1s and 5187 G0s with data on every metabolite on every occasion (Additional files 5, 6, 7, 8, 9, 10, 11, 12: Supplementary Tables 4, 5, 6, 7, 8, 9, 10, 11), with expectedly lower estimate precision.

## Discussion

In this study, we examined sex differences in systemic metabolites at multiple life stages, from childhood to middle adulthood, to help identify circulating traits that may underpin higher age-adjusted CHD rates commonly seen among males. Our results suggest that, from adolescence onwards, lipids (particularly triglycerides) in VLDL are higher among males while levels of other CHD-related traits including LDL cholesterol, apolipoprotein-B, and inflammatory glycoprotein acetyls, are higher among females. These patterns of effect were highly consistent when adjusting for BMI measured on each occasion. Causal analyses of these traits in relation to clinical endpoints are needed to understand whether they differentially affect CHD risk among males and females.

A causal role for LDL cholesterol in CHD aetiology is strongly supported by human genetic [35, 36] and pharmacological intervention [37, 38] studies. Recent genetic evidence also supports a role of higher apolipoprotein-B in CHD independent of LDL cholesterol concentration [39–42]. Despite this, our results suggest that differences in absolute levels of LDL cholesterol or apolipoprotein-B are unlikely to underpin the higher risk of CHD experienced among males, since levels were lower (more favourable) among males in adolescence and young adulthood among G1s, as well as in middle adulthood among G0s. However, this does not exclude the possibility of LDL cholesterol, apolipoprotein-B, or other metabolites which did not differ by sex in these analyses having a differential effect on CHD among males and females; sex-stratified causal analyses of these in relation to CHD itself are needed to determine this. The present study aimed to estimate absolute effects of sex on metabolites, not relative effects of metabolites on CHD by sex.

In contrast, our results suggest that triglyceride content—particularly in VLDL—are higher (more adverse) among males in adolescence and that this sex difference is larger in young adulthood and larger still in middle adulthood. The tendency for males to have higher circulating triglycerides has been observed previously [14, 15], but how differences relative to females progress across multiple life stages has been unclear due to lack of repeated measures. Higher triglycerides among males at multiple life stages observed here, together with previous genetic evidence of a causal role of triglycerides for CHD [40, 43], support triglycerides as a key target for CHD prevention, particularly among males. Whether triglycerides increase CHD risk more greatly among males requires sex-stratified analyses.

Equally strong tendencies were found for lower HDL cholesterol among males than females at later life stages. However, despite robust observational associations of lower HDL cholesterol with higher CHD risk [44], genetic [35, 45] and pharmacological intervention studies [46] do not support a causal effect of HDL cholesterol on CHD. Nevertheless, HDL cholesterol is increasingly regarded as a useful non-causal marker for insulin resistance and other traits that are causal, namely circulating triglycerides, with which it tracks strongly in opposing directions [44, 47]. The utility of HDL cholesterol as a marker of pre-glycaemic changes is further supported by results here showing progressively lower HDL cholesterol levels to coincide with progressively higher branched chain amino acid levels, which are likely components of early-stage insulin resistance [48].

The earlier measurement occasions used in this study (8 years and 16 years) spanned puberty for most participants—an important period of growth and development. The mean age at puberty onset here was estimated at 13.6 years for males and 11.7 years for females based on growth-curve modelling of repeated height measures. This transition profoundly influences physical and sexual maturity [49], and among females results in the release of oestrogen and other sex hormones which are thought to result in less adverse lipid profiles [15]; whether testosterone release among males itself results in more adverse lipid profiles is less clear [15, 50]. Potential benefits of oestrogen or harms of testosterone on lipid profiles are supported by present results which suggest that total lipids and triglycerides in VLDL are lower among males before puberty, but then switch direction after puberty onset to become higher among males in adolescence and young adulthood, a difference which is even greater in middle adulthood. This was also true of cholesterol in HDL but in the reverse direction (higher levels among males before puberty, then lower levels after puberty). Together, this supports puberty as a pivotal time for the emergence of life-long sex differences in triglycerides and risk-marking HDL cholesterol.

How these sex differences extend beyond middle age, when the menopausal transition is complete, is less clear. Females in middle adulthood were measured here at mean age 47.9 years, when natural menopause has either not yet begun or is typically in early stages. Menopause status was examined here using the rigorous STRAW criteria [18, 28], and sex differences in metabolites were re-examined when including only those females who were pre-menopause; these indicated similar results as seen when all females were included. Sex differences were then re-examined when including only those females who were post-menopause (also excluding those who were peri-menopause), and sex differences in VLDL lipids appeared narrower than were seen when all females were included. In contrast, sex differences in IDL, LDL, and HDL lipids appeared wider. Differences also appeared narrower in branched chain amino acids, but wider in most other metabolites including fatty acids and glucose. This suggests that proposed cardio-protective effects of female oestrogen release [14, 15], which would be reduced post-menopause [18, 28], are VLDL-specific, but further studies in females with natural and surgical menopause are needed to confirm this.

### Limitations

The limitations of this study include modest sample sizes among G1s, particularly for complete case analyses, and greater loss to follow-up among males. Unequal numbers of males and females may result in biased estimates of sex differences if loss to follow-up is related to both sex and outcomes; however, most characteristics were comparable between included and excluded males and females, suggesting that such bias is unlikely or small. Estimates were based on serial cross-sectional associations from linear regression models which did not account for correlation between repeated measures of metabolites over time. In the current sample, the moderate correlations between repeated measures of select metabolites tended to weaken with increasing follow-up time, e.g. the Pearson correlation between VLDL triglycerides measured at 8 years and 16 years, 8 years and 18 years, and 8 years and 25 years was 0.34, 0.33, and 0.25, respectively. For LDL cholesterol, the correlations for these same time periods were 0.61, 0.57, and 0.46; for apolipoprotein-B, these were 0.57, 0.51, and 0.43; and for glycoprotein acetyls, these were 0.31, 0.26, and 0.22. Mixed modelling was not presently feasible given the volume of traits examined, the sparsity of repeated measures for reliable non-linear modelling, and substantial occasion-level variability in several metabolite ratios. The development of a uniform approach for modelling trajectories under such conditions will improve estimates in future. Data were of a unique multi-generational nature, comprising offspring and their parents; this opens the possibility of additional sources of bias from cohort and period effects. Selection bias could also be differential between G1s and G0s. This is indicated by higher proportions of maternal smoking among included G1s than among included G0s who subsequently participated in clinic assessments several years after pregnancy, indicating that mothers who attended clinic assessments were a relatively healthy subset of those initially recruited.

We examined metabolites from a targeted NMR platform which is comprised of several traits considered a priori to be etiologically important for CHD, such as cholesterol and triglyceride content in various non-HDL particle types. However, this platform does not capture other potentially important factors like insulin, sex hormones, regulatory proteins, or different inflammatory traits. Examining additional metabolites measured using untargeted mass-spectrometry (MS) could reveal more detailed and novel sex differences. Examining circulating proteins from novel proteomic platforms could also be advantageous and help identify traits which are potentially more readily targetable via drugs. Such data are not currently available at scale or with repeated measures at different life stages.

Biological sex is an essentially randomised exposure within a causal inference framework with no expected influence of common causes (confounders), although such factors could influence study participation. Numerous explanations exist for sex differences in metabolites described here; these would largely be considered mediators. Estimates generated here therefore pertain to total effects of sex (male vs female), rather than direct effects of sex which are independent of potential mediating factors. In additional analyses, we adjusted for BMI measured at the time of metabolite assessment because biological sex is known to influence BMI [7, 12] and BMI is supported by several observational and MR studies as influencing these same metabolites [29–32]. This adjustment did not appear to substantially attenuate effect estimates for sex, suggesting that adiposity may not mediate/underpin the sex differences in metabolites observed here. Numerous potential lifestyle-related factors could still have mediating roles, such as adverse dietary patterns, smoking behaviour, or alcohol consumption. Future studies could examine these pathways using formal mediation analyses; although such lifestyle-related factors carry the added challenge of high measurement error and variability with time, with changing prevalence and potentially changing metabolic impacts across the life course.

## Conclusions

Measures of systemic metabolites at multiple life stages suggest that VLDL lipids (particularly triglycerides) are higher among males relative to females in adolescence and that this difference is larger at older ages. In contrast, other CHD-related metabolites including LDL cholesterol, apolipoprotein-B, and inflammatory glycoprotein acetyls are higher among females in adolescence, with similar patterns with advancing age. Such life course trends may inform causal analyses with clinical endpoints in specifying traits which underpin higher age-adjusted CHD rates commonly seen among males.

## Supplementary Information

**Additional file 1.** Supplementary Methods.

**Additional file 2: Supplementary Table 1.** Descriptive characteristics of ALSPAC participants eligible vs ineligible for ≥1 analysis.

**Additional file 3: Supplementary Table 2.** Descriptive characteristics of participating ALSPAC mothers based on partners’ participation status.

**Additional file 4: Supplementary Table 3.** Descriptive characteristics of participating ALSPAC offspring based on partners’ (of mothers) participation status.

**Additional file 5: Supplementary Table 4.** Sex differences in metabolites at different life stages among ALSPAC offspring and parents.

**Additional file 6: Supplementary Table 5.** Sex differences in metabolites at different life stages among ALSPAC offspring and parents.

**Additional file 7: Supplementary Table 6.** Sex differences in metabolites at different life stages among ALSPAC offspring and parents, with BMI adjustment.

**Additional file 8: Supplementary Table 7.** Sex differences in metabolites at different life stages among ALSPAC offspring and parents, with BMI adjustment.

**Additional file 9: Supplementary Table 8.** Sex differences in metabolites in middle adulthood among ALSPAC parents, with menopausal comparisons.

**Additional file 10: Supplementary Table 9.** Sex differences in metabolites in middle adulthood among ALSPAC parents, with menopausal comparisons.

**Additional file 11: Supplementary Table 10.** Sex differences in metabolites in middle adulthood among ALSPAC parents, with menopausal comparisons and BMI adjustment.

**Additional file 12: Supplementary Table 11.** Sex differences in metabolites in middle adulthood among ALSPAC parents, with menopausal comparisons and BMI adjustment.

## Data Availability

Individual-level ALSPAC data are available following an application. This process of managed access is detailed at www.bristol.ac.uk/alspac/researchers/access. Cohort details and data descriptions for ALSPAC are publicly available at the same web address.
