# Supplementary material for: Sex differences in systemic metabolites at four life stages: cohort study with repeated metabolomics
Source: BMC Med. 2021 Feb 24;19:58. doi: 10.1186/s12916-021-01929-2 (PMC7903597; doi:10.1186/s12916-021-01929-2)
Supplement: Supplementary file 1 — Additional file 1. Supplementary Methods. [file 12916_2021_1929_MOESM1_ESM.docx]

**Supplementary Methods**

*Participant characteristics used for sample description*

Highest level of maternal and partner education at the time of offspring birth was based on a questionnaire completed by the mother about herself and about her partner, both grouped as ‘Certificate of Secondary Education (CSE)’ (exams taken in different subjects usually at age 16y, which was the minimum school leaving age at the time), ‘vocational’, ‘Ordinary (O)-level’ (exams taken in different subjects usually at age 16y that were considered more academic than CSE or vocational qualifications), ‘Advanced (A)-level’ (usually taken at age 18y), or ‘university degree’.

Maternal smoking during pregnancy was defined as having self-reported smoking any type of tobacco at any time during pregnancy based on repeated questionnaires at each trimester. Partner smoking was defined as the mother having reported on behalf of the partner that the partner currently smokes any type of tobacco based on a questionnaire during the first trimester.

Among offspring and parents at each clinic, height was measured in light clothing without shoes to the nearest 0.1 cm using a Harpenden stadiometer and weight was measured to the nearest 0.1 kg using a Tanita scale. Body mass index (BMI) was then calculated as weight (kg) / height (m)^2^. Pre-pregnancy BMI of the mother was also calculated based on recalled height and weight at the beginning of her pregnancy (referring to the time immediately before starting her pregnancy). BMI of the partner at the start of the pregnancy was also calculated based on the partner’s reported height and weight at that time.

Among male and female offspring, puberty timing was estimated via age at peak height velocity (i.e. the growth spurt) based on SuperImposition by Translation And Rotation (SITAR) growth curve modelling (18) of up to 10 height measurements from age 5y to 20y (detailed previously (25)). This measure correlates highly with more direct measures of secondary sexual characteristics (26) and has the important advantage of being relevant and comparably well measured among males and females.

Among mothers, at the time cardiometabolic traits were measured, a range of information about the menstrual cycle was collected, comprising whether the women had experienced a menstrual period in the last 3 months and 12 months, the regularity of their cycle, the frequency of their periods and the date of their last menstrual period. Menopausal status at the time metabolic traits were measured was determined via questionnaire using the Stages of Reproductive Age Workshop (STRAW) criteria (detailed previously (27, 28)) to produce 6 mutually exclusive categories of ‘reproductive’, ‘menopause transition’, ‘first year post-menopause’, ‘second year post-menopause’, ‘third to sixth year post-menopause’ and ‘late post-menopause’. These 6 categories were then collapsed into 3 general categories for the present purposes of description and supplementary analysis: ‘pre-menopause’, ‘peri-menopause’ (menopause transition), or ‘post-menopause’ (≥ 1 year post-menopause).

*Supplementary analyses*

The main analyses of sex differences in G0 parents (when mean age was 47.9y among mothers and 53.3y among partners) include females who are predominantly pre-menopause (62.1% of females reported current menstrual periods). To better examine the impact of natural menopause on results, we re-examined sex differences in cardiometabolic traits among G0 parents when including, first: only those females who were considered to be pre-menopause; and second: only those females who were considered to be post-menopause (i.e. ≥ 1 year post-menopause and neither pre-menopause nor peri-menopause as defined as menopause transition). Both analyses excluded females who reported having either a hysterectomy, oophorectomy, ablation/resection, chemotherapy or radiation therapy (i.e. surgical menopause), use of hormonal contraception, or use of hormone replacement (27, 28).

All models were repeated on a complete case sample of 769 G1 offspring (46% male) and 5,187 G0 parents (19% male) who had complete data on sex, age, and every cardiometabolic trait on every occasion. This was done to ensure that results were not driven by the changing sample across time points.
